# Oxygen targets following cardiac arrest: A meta-analysis of randomized controlled trials

**DOI:** 10.1016/j.ijcha.2023.101243

**Published:** 2023-07-05

**Authors:** Huzaifa Ahmad Cheema, Arman Shafiee, Amirhossein Akhondi, Niloofar Seighali, Abia Shahid, Mohammad Ebad Ur Rehman, Talal Almas, Sebastian Hadeed, Abdulqadir J. Nashwan, Soban Ahmad

**Affiliations:** a Department of Cardiology, King Edward Medical University, Lahore, Pakistan; b Clinical Research Development Unit, Alborz University of Medical Sciences, Karaj, Iran; c Student Research Committee, School of Medicine, Alborz University of Medical Sciences, Karaj, Iran; d Department of Medicine, Rawalpindi Medical University, Rawalpindi, Pakistan; e Department of Cardiovascular Medicine, Galway University Hospital, Galway, Ireland; f Department of Internal Medicine, Royal College of Surgeons in Ireland, Dublin, Ireland; g Hamad Medical Corporation, Doha, Qatar; h Department of Internal Medicine, East Carolina University, Greenville, NC, USA

**Keywords:** Oxygen, Hyperoxia, Cardiac arrest, Meta-analysis

## Abstract

**Introduction:**

The appropriate oxygen target post-resuscitation in out-of-hospital cardiac arrest (OHCA) patients is uncertain. We sought to compare lower versus higher oxygen targets in patients following OHCA.

**Methods:**

We searched MEDLINE, Embase, the Cochrane Library, and ClinicalTrials.gov until January 2023 to include all randomized controlled trials (RCTs) that evaluated conservative vs. liberal oxygen therapy in OHCA patients. Our primary outcome was all-cause mortality at 90 days while our secondary outcomes were the level of neuron-specific enolase (NSE) at 48 h, ICU length of stay (LOS), and favorable neurological outcome (the proportion of patients with Cerebral Performance Category scores of 1–2 at end of follow-up). We used RevMan 5.4 to pool risk ratios (RRs) and mean differences (MDs).

**Results:**

Nine trials with 1971 patients were included in our review. There was no significant difference between the conservative and liberal oxygen target groups regarding the rate of all-cause mortality (RR 0.95, 95% CI: 0.80 to 1.13; I^2^ = 55%). There were no significant differences between the two groups when assessing favorable neurological outcome (RR 1.01, 95% CI: 0.92 to 1.10; I^2^ = 4%), NSE at 48 h (MD 0.04, 95% CI: −0.67 to 0.76; I^2^ = 0%), and ICU length of stay (MD −2.86 days, 95% CI: −8.00 to 2.29 days; I^2^ = 0%).

**Conclusions:**

Conservative oxygen therapy did not decrease mortality, improve neurologic recovery, or decrease ICU LOS as compared to a liberal oxygen regimen. Future large-scale RCTs comparing homogenous oxygen targets are needed to confirm these findings.

## 1 Introduction

Cardiac arrest often results in hypoxic-ischemic brain injury with less than 10% of patients achieving meaningful neurologic recovery [1]. Comatose patients following out-of-hospital cardiac arrest (OHCA) frequently require mechanical ventilation with supplemental oxygen. However, there is equipoise regarding the optimal oxygen target in such patients. Studies have shown that hyperoxia might cause exacerbation of neurological injury by producing excessive oxygen free radicals [2], [3]. Moreover, liberal oxygenation has also been associated with an increased risk of ischemic encephalopathy and death [4]. On the other hand, conservative oxygen therapy may be further detrimental to already hypoxic tissue. Therefore, determining an appropriate oxygen target post-resuscitation in OHCA patients is of paramount importance. Recently, there has been an increased focus on different suggested oxygen targets in randomized clinical trials (RCTs), with the largest of these trials published recently [5]. Hence, the present paper sought to review the current literature and conduct a contemporary meta-analysis to compare lower versus higher oxygen targets in patients following OHCA.

## 2 Methods

We registered our protocol with PROSPERO (CRD42022383931) and conducted this meta-analysis following the guidance presented in the Cochrane Handbook for Systematic Reviews of Intervention. We performed a systematic search on MEDLINE (Ovid), the Cochrane Library, Embase, and ClinicalTrials.gov from inception to January 2023 to retrieve relevant studies. Additionally, we manually searched the reference lists of relevant studies. The detailed search strategy is given in Supplementary Table 1. The screening process was carried out independently by two authors based on the following inclusion criteria: (1) population: adults with OHCA; (2) interventions: conservative oxygen therapy versus liberal oxygen therapy as defined by the individual RCTs; and 3) type of study: RCTs only. We defined all-cause mortality at 90 days as our primary outcome. If a study did not report 90-day mortality, we used the endpoint closest to it. Our secondary outcomes were: (1) the level of neuron-specific enolase (NSE) at 48 h; (2) ICU length of stay (LOS); and (3) favorable neurological outcome: the proportion of patients with Cerebral Performance Category (CPC) scores of 1–2 at end of follow-up.

**Table 1:** Characteristics of the included trials.

**ID**	**Author**	**Year**	**Country**	**RCT name**	**Population**	**Total patients**	**Age**	**Follow up**	**O2 target (mm Hg) (Conservative (restrictive) vs. liberal oxygen therapy)**	**Risk of bias**
1	Schmidt et al.	2022	Denmark	BOX	Comatose adults with out-of-hospital cardiac arrest	789	Restrictive Oxygen Target group: 62 ± 13Liberal Oxygen Target group: 63 ± 14	90 days	Restrictive: 9 to 10 kPa (68 to 75 mm Hg) / liberal: 13 to 14 kPa (98 to 105 mm Hg)	Low
2	Schjørring et al.	2021	Denmark	HOT-ICU	Adult patients who had recently been admitted to the ICU	2928: 335 cardiac arrest patients	NA	90 days	A Pao2 of 60 mm Hg (lower-oxygenation group) or a Pao2 of 90 mm Hg (higher-oxygenation group)	Some concerns
3	Jakkula et al.	2018	Finland	COMACARE	Adult unconscious, mechanically ventilated patients, resuscitated from witnessed out-of-hospital cardiac arrest	120	Normoxia group (59 ± 13), Moderate hyperoxia group (60 ± 14)	180 days	Arterial oxygen tension [PaO2] 10–15 kPa) or moderate hyperoxia (PaO2 20–25 kPa	Some concerns
4	Bray et al.	2018	Australia	EXACT pilot trial	Adults (age ≥ 18 years), unconscious (Glasgow Coma Scale < 9) with an advanced airway (endotracheal tube or supraglottic airways) in situ and sustained ROSC following an out-of-hospital cardiac arrest.	61	Mean +/- SD: 2–4 L/min(case): 64 ± 13.5 ≥10 L/min (non-case): 60.5 ± 9	July 2015 - May 2017	2–4 L/min vs. ≥ 10 L/min	Some concerns
5	Paul Young	2014	New Zealand	–	Patients who were ventilated via a laryngeal mask airway or endotracheal with an estimated age 16–90 and had return of spontaneous circulation following out-of-hospital cardiac arrest due to a suspected primary cardiac cause with an initialrhythm of VF or VT.	18	Standard oxygen 61.4 ± 20.8 titrated oxygen 71.6 ± 10.7	Between 13/10/2012 and21/09/2013	SpO2 > 95% (standard group) vs. SpO2 of 90–94%	Some concerns
6	Diane Mackle	2019	New Zealand	ICU-ROX	Adult patients who were anticipated to require mechanical ventilation beyond the day after recruitment in the ICU to receive conservative or usual oxygen therapy	1000: 166 with suspected hypoxic-ischemic encephalopathy	Conservative 58.1 ± 16.2usual 57.5 ± 16.1	28 days after randomization	Usual Oxygen Therapy (spO2 ≥ 91%) vs.Conservative Oxygen Therapy (91%≤ spO2 < 97%)	Some concerns
7	Kuisma, M.	2006	Finland	–	Patients with a bystander witnessed out-of-hospital ventricular fibrillation treated by the mobile intensive care unit, and if a return of spontaneous circulation was sustained for less than 60 min, the patient was excluded from the study.	28	30% oxygen for 60 min: 61.9 ± 13.6 100% oxygen for 60 min:64.3 ± 7.8	Patients were followed until in-hospital death or survival to hospital discharge	Group A (ventilated with 30% of oxygen) vs.group B (ventilated with 100 % of oxygen)	Some concerns
8	Thomas, M.	2019	United Kingdom	PROXY	Patients were 18 years or older and had an out-of-hospital cardiac arrest	35	(Got 100% oxygen) 64(Got titrated oxygen) 70	90 days	Titrated O_2_ (94–98%) vs. 100% O_2_ therapy	Some concerns
9	Bernard	2022	Australia	EXACT	Adults (age ≥ 18 years), unconscious after return of spontaneous circulation with an advanced airway (endotracheal tube or supraglottic airways), Spo2 of at least 95% while receiving more than 10 L/min of oxygen or Fio2 of 100%, and transport planned to a participating hospital.	425	65.5 (53.1–76.4)	12 months	Spo_2_ of 90% to 94% vs Spo_2_ of 98% to 100%	Low

We assessed the risk of bias for each trial using the Cochrane Risk of Bias version 2 (RoB 2.0) tool. We used a random-effects model to pool risk ratios (RRs) for dichotomous outcomes and mean differences (MDs) for continuous outcomes with their corresponding 95% confidence intervals (CIs). We chose the random-effects model because we anticipated our included studies to be considerably heterogeneous. The I^2^ statistic was used to evaluate heterogeneity. All statistical analyses were performed using R software version 4.1.0 (the meta package) [6]. We were unable to assess publication bias as the number of included studies was less than 10.

## 3 Results

Nine trials with 1971 patients were included in our review [2], [3], [5], [7], [8], [9], [10], [11], [12]. The detailed selection process is depicted in a PRISMA flowchart (Supplementary Fig. 1) The detailed characteristics of each trial are presented in Table 1. All trials were ascertained to have some concerns of bias except two which were rated to be at low risk of bias (Supplementary Figure 2) [5], [12].

**Fig. 1:**
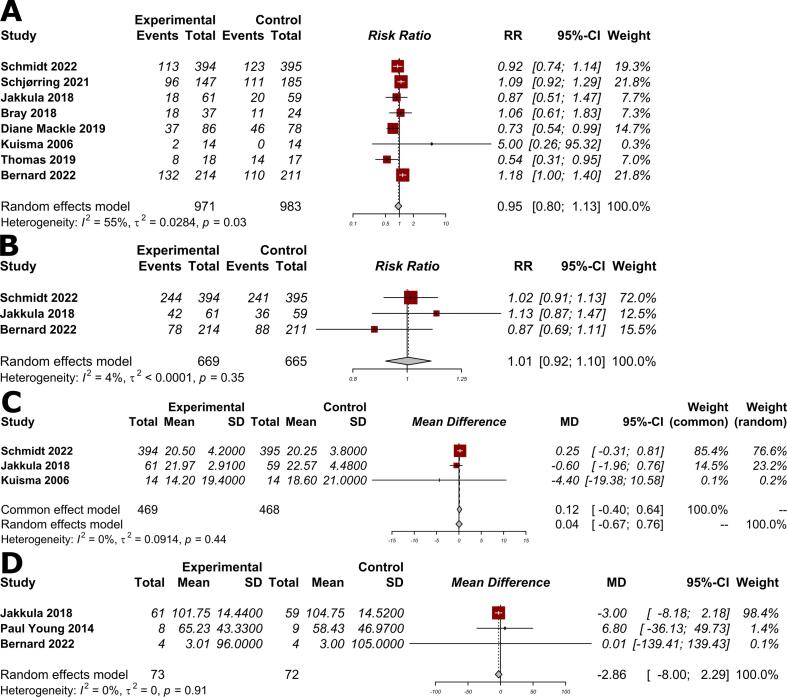
Effect of conservative oxygen therapy versus liberal oxygen therapy on: A) all-cause mortality; B) good neurological outcome; C) levels of neuron-specific enolase at 48 h; and D) length of ICU stay.

The pooled results from eight studies did not show a significant difference between the conservative and liberal oxygen target groups regarding the rate of all-cause mortality (RR 0.95, 95% CI: 0.80 to 1.13; I^2^ = 55%; Fig. 1A). There were no significant differences between the two groups when assessing favorable neurological outcome (RR 1.01, 95% CI: 0.92 to 1.10; I^2^ = 4%; Fig. 1B), NSE at 48 h (MD 0.04, 95% CI: −0.67 to 0.76; I^2^ = 0%; Fig. 1C), and ICU LOS (MD −2.86 days, 95% CI: −8.00 to 2.29 days; I^2^ = 0%; Fig. 1D).

## 4 Discussion

To the best of our knowledge, this is the largest meta-analysis on this topic to date. In this study, we observed no difference between conservative oxygen therapy and liberal oxygen therapy regarding mortality or any other studied clinical outcome.

The idea that proposed significant adverse effects of high oxygen therapy was first derived from experimental studies [13]. A meta-analysis on animal trials by Pilcher et al. showed worse neurological outcomes after the administration of 100% oxygen compared to the group which received restrictive oxygen therapy [13]. These findings were further corroborated in humans by observational studies [14]. However, evidence from RCTs is needed to make any explicit recommendations for clinical practice.

Until 2020, several small RCTs had been performed. An individual-level patient data meta-analysis of RCTs published by Young et al. showed a significant reduction in mortality with conservative oxygen therapy after pooling data from 429 patients [15]. This meta-analysis did not include the data from HOT-ICU [9], EXACT [12], and the largest trial to date on this topic which has been recently published by the investigators of the BOX trial [5]. In our meta-analysis, after pooling the results from these trials, we found no benefit of conservative oxygen therapy in cardiac arrest patients. Our results suggest that the focus needs to be shifted to therapeutic interventions other than different oxygen, blood pressure, and carbon dioxide targets as these measures have shown no benefits [16], [17].

Our study has several strengths. This is the largest meta-analysis to date including the data of 1971 patients from 9 RCTs. Moreover, our outcomes had low heterogeneity. The main limitation of our study was the variability of the study design, follow-up periods, and outcome definitions of the included trials.

In conclusion, conservative oxygen therapy did not decrease mortality, improve neurologic recovery, or decrease ICU LOS as compared to a liberal oxygen regimen. Future large-scale RCTs comparing homogenous oxygen targets are needed to confirm these findings.

Statements and Declarations.

Financial support.

No financial support was received for this study.

## Declaration of Competing Interest

The authors declare that they have no known competing financial interests or personal relationships that could have appeared to influence the work reported in this paper.
