# Supplementary material for: Oxygen targets following cardiac arrest: A meta-analysis of randomized controlled trials
Source: Int J Cardiol Heart Vasc. 2023 Jul 5;47:101243. doi: 10.1016/j.ijcha.2023.101243 (PMC10359856; doi:10.1016/j.ijcha.2023.101243)

**Table S1. Search strategy for different databases.**

| **MEDLINE** |
| --- |
| 1 hyperoxia.mp. or exp Hyperoxia/ |
| 2 hyperoxemia.mp. |
| 3 hyperoxygenation.mp. |
| 4 overoxygenation.mp. |
| 5 (permissive adj5 hypoxia).mp. [mp=title, abstract, original title, name of substance word, subject heading word, keyword heading word, protocol supplementary concept word, rare disease supplementary concept word, unique identifier, synonyms] |
| 6 (permissive adj5 hypoxemia).mp. [mp=title, abstract, original title, name of substance word, subject heading word, keyword heading word, protocol supplementary concept word, rare disease supplementary concept word, unique identifier, synonyms] |
| 7 normoxia.mp. |
| 8 normoxemia.mp. |
| 9 liberal*.tw. |
| 10 conservative*.tw. |
| 11 conventional*.tw. |
| 12 1 or 2 or 3 or 4 or 5 or 6 or 7 or 8 or 9 or 10 or 11 |
| 13 exp Oxygen Inhalation Therapy/ or exp Oxygen/ |
| 14 (supplement* adj5 oxygen*).mp. [mp=title, abstract, original title, name of substance word, subject heading word, keyword heading word, protocol supplementary concept word, rare disease supplementary concept word, unique identifier, synonyms] |
| 15 (oxygen* adj5 therap*).mp. [mp=title, abstract, original title, name of substance word, subject heading word, keyword heading word, protocol supplementary concept word, rare disease supplementary concept word, unique identifier, synonyms] |
| 16 (inhal* adj5 oxygen*).mp. [mp=title, abstract, original title, name of substance word, subject heading word, keyword heading word, protocol supplementary concept word, rare disease supplementary concept word, unique identifier, synonyms] |
| 17 (inspir* adj5 oxygen*).mp. [mp=title, abstract, original title, name of substance word, subject heading word, keyword heading word, protocol supplementary concept word, rare disease supplementary concept word, unique identifier, synonyms] |
| 18 fio2.mp. |
| 19 13 or 14 or 15 or 16 or 17 or 18 |
| 20 exp Randomized Controlled Trial/ |
| 21 randomized controlled trial.pt. |
| 22 controlled clinical trial.pt. |
| 23 random*.tw. |
| 24 placebo.ab. |
| 25 drug therapy.fs. |
| 26 trial.ab. |
| 27 groups.ab. |
| 28 20 or 21 or 22 or 23 or 24 or 25 or 26 or 27 |
| 29 exp Hospital Mortality/ or mortality.mp. or exp Mortality/ |
| 30 exp Survival/ or exp Survival Rate/ or survival.mp. or exp Survival Analysis/ |
| 31 disability.mp. or exp Disability Evaluation/ |
| 32 exp Cognition Disorders/ |
| 33 exp Death/ |
| 34 death*.tw. |
| 35 died.tw. |
| 36 29 or 30 or 31 or 32 or 33 or 34 or 35 |
| 37 12 and 19 |
| 38 28 and 36 |
| 39 37 and 38 |
| 40 exp Pregnancy/ |
| 41 exp Obstetrics/ |
| 42 pregna*.tw. |
| 43 obstetric*.tw. |
| 44 mice.tw. |
| 45 mouse.tw. |
| 46 cell*.tw. |
| 47 vitro.tw. |
| 48 rodent*.tw. |
| 49 rat.tw. |
| 50 rats.tw. |
| 51 pediatric*.tw. |
| 52 40 and 41 and 42 and 43 and 44 and 45 and 46 and 47 and 48 and 49 and 50 and 51 |
| 53 39 not 52 |
| 54 limit 53 to (animals or pregnancy) |
| 55 53 not 54 |
| 56 limit 55 to ("all infant (birth to 23 months)" or "newborn infant (birth to 1 month)" or "infant (1 to 23 months)" or "preschool child (2 to 5 years)" or "child (6 to 12 years)") |
| 57 55 not 56 |
| **EMBASE** |
| 1 hyperoxia.mp. or exp hyperoxia/ |
| 2 hyperoxemia.mp. |
| 3 hyperoxygenation.mp. |
| 4 overoxygenation.mp. |
| 5 (permissive adj5 hypoxia).mp. [mp=title, abstract, heading word, drug trade name, original title, device manufacturer, drug manufacturer, device trade name, keyword, floating subheading] |
| 6 (permissive adj5 hypoxemia).mp. [mp=title, abstract, heading word, drug trade name, original title, device manufacturer, drug manufacturer, device trade name, keyword, floating subheading] |
| 7 normoxia.mp. |
| 8 normoxemia.mp. |
| 9 liberal*.tw. |
| 10 conservative*.tw. |
| 11 conventional*.tw. |
| 12 1 or 2 or 3 or 4 or 5 or 6 or 7 or 8 or 9 or 10 or 11 |
| 13 exp oxygen therapy/ or exp oxygen/ |
| 14 (supplement* adj5 oxygen*).mp. [mp=title, abstract, heading word, drug trade name, original title, device manufacturer, drug manufacturer, device trade name, keyword, floating subheading] |
| 15 (oxygen* adj5 therap*).mp. [mp=title, abstract, heading word, drug trade name, original title, device manufacturer, drug manufacturer, device trade name, keyword, floating subheading] |
| 16 (inhal* adj5 oxygen*).mp. [mp=title, abstract, heading word, drug trade name, original title, device manufacturer, drug manufacturer, device trade name, keyword, floating subheading] |
| 17 (inspir* adj5 oxygen*).mp. [mp=title, abstract, heading word, drug trade name, original title, device manufacturer, drug manufacturer, device trade name, keyword, floating subheading] |
| 18 fio2.tw. |
| 19 13 or 14 or 15 or 16 or 17 or 18 |
| 20 randomized controlled trial/ |
| 21 randomized controlled trial.pt. |
| 22 controlled clinical trial.pt. |
| 23 random*.tw. |
| 24 placebo.ab. |
| 25 drug therapy.fs. |
| 26 trial.ab. |
| 27 groups.ab. |
| 28 20 or 21 or 22 or 23 or 24 or 25 or 26 or 27 |
| 29 exp mortality/ or exp cardiovascular mortality/ or exp mortality rate/ or mortality risk/ or exp surgical mortality/ or mortality.mp. or exp hospital mortality/ or exp standardized mortality ratio/ |
| 30 exp survival analysis/ or exp survival rate/ or survival.mp. or exp survival/ or exp median survival time/ or exp cause specific survival/ or exp survival time/ or exp short term survival/ or exp long term survival/ or exp overall survival/ |
| 31 exp "International Classification of Functioning, Disability and Health"/ or exp Roland Morris disability questionnaire/ or exp disability severity/ or exp language disability/ or exp disability/ or exp Sheehan Disability Scale/ or exp Expanded Disability Status Scale/ or exp ADL disability/ or disability.mp. or exp physical disability/ |
| 32 exp cognitive defect/ |
| 33 exp death/ or exp sudden death/ or exp "time of death"/ or exp sudden cardiac death/ |
| 34 death*.tw. |
| 35 died.tw. |
| 36 exp brain hypoxia/ or exp heart arrest/ |
| 37 29 or 30 or 31 or 32 or 33 or 34 or 35 or 36 |
| 38 exp pregnancy/ |
| 39 exp obstetrics/ |
| 40 pregna*.tw. |
| 41 obstetric*.tw. |
| 42 mice.tw. |
| 43 mouse.tw. |
| 44 cell*.tw. |
| 45 vitro.tw. |
| 46 rodent*.tw. |
| 47 rat.tw. |
| 48 rats.tw. |
| 49 pediatric*.tw. |
| 50 neonat*.tw. |
| 51 newborn/ |
| 52 38 or 39 or 40 or 41 or 42 or 43 or 44 or 45 or 46 or 47 or 48 or 49 or 50 or 51 |
| 53 12 and 19 and 28 and 37 |
| 54 53 not 52 |
| 55 limit 54 to (animals or animal studies) |
| 56 54 not 55 |
| 57 limit 56 to (embryo <first trimester> or infant <to one year> or preschool child <1 to 6 years> or school child <7 to 12 years>) |
| 58 56 not 57 |
| **Cochrane Central Register of Controlled Trials (CENTRAL)** |
| #1 hyperoxia |
| #2 MeSH descriptor: [Hyperoxia] explode all trees |
| #3 hyperoxemia |
| #4 hyperoxygenation |
| #5 MeSH descriptor: [Oxygen Inhalation Therapy] explode all trees |
| #6 (neonate):ti,ab, kw |
| #7 MeSH descriptor: [Infant, Newborn] explode all trees |
| #8 (paediatrics):ti,ab, kw |
| #9 MeSH descriptor: [Pediatrics] explode all trees |
| #10 MeSH descriptor: [Pregnancy] explode all trees |
| #11 (pregnancy):ti,ab,kw |
| #12 (exercise):ti,ab,kw |
| #13 (training):ti,ab,kw |
| #14 (mice):ti,ab,kw |
| #15 (rat):ti,ab,kw |
| #16 MeSH descriptor: [Exercise] explode all trees |
| #17 #6 or #7 or #8 or #9 or #10 or #11 or #12 or #13 or #14 or #15 or #16 |
| #18 (#1 or #2 or #3 or #4) and (#5) |
| #19 #18 not #17 |

**Supplementary Figure 1.** PRISMA 2020 flowchart of study selection process.

**
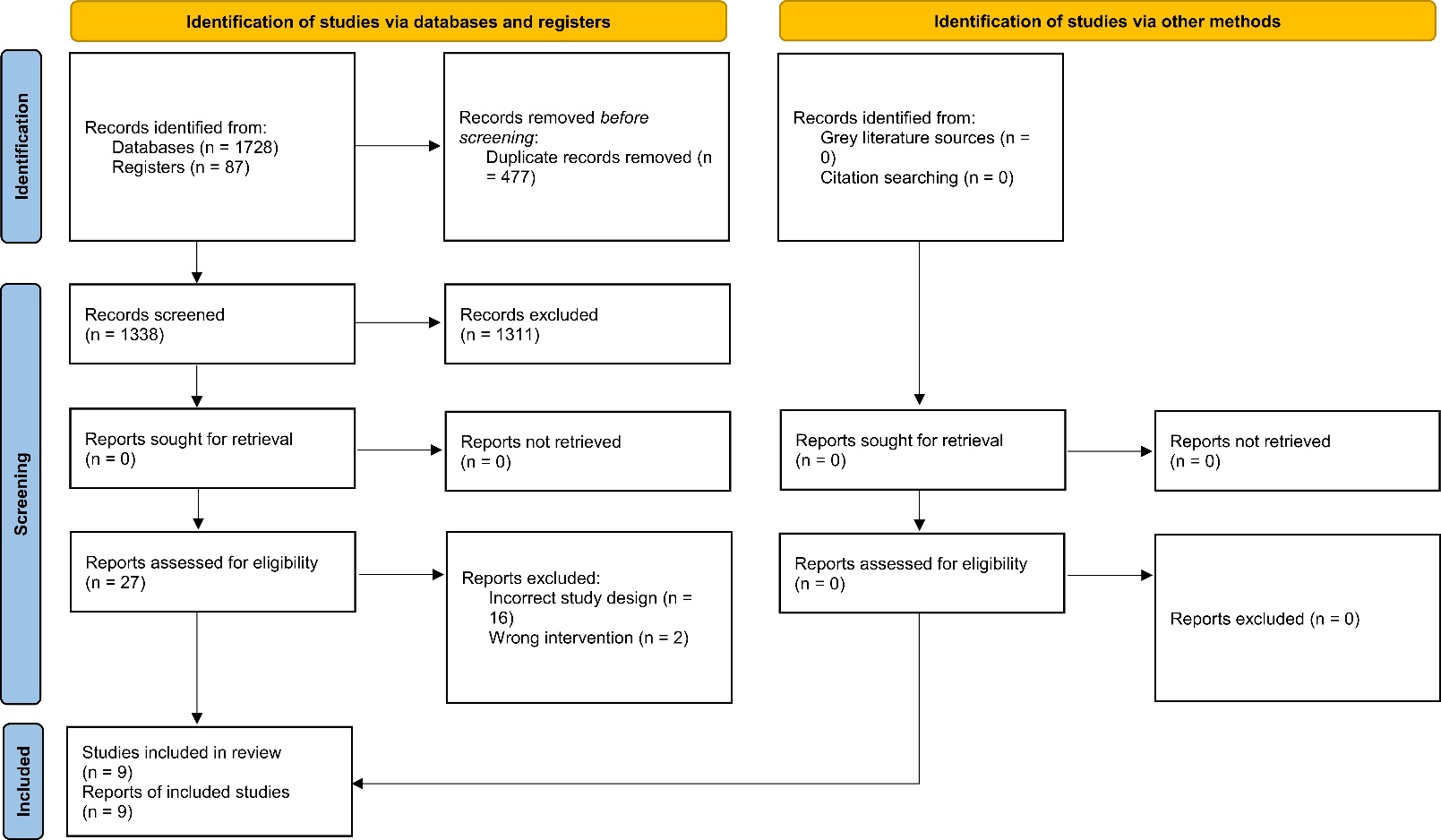
**

**Supplementary Figure 2.** Quality assessment of included RCTs.


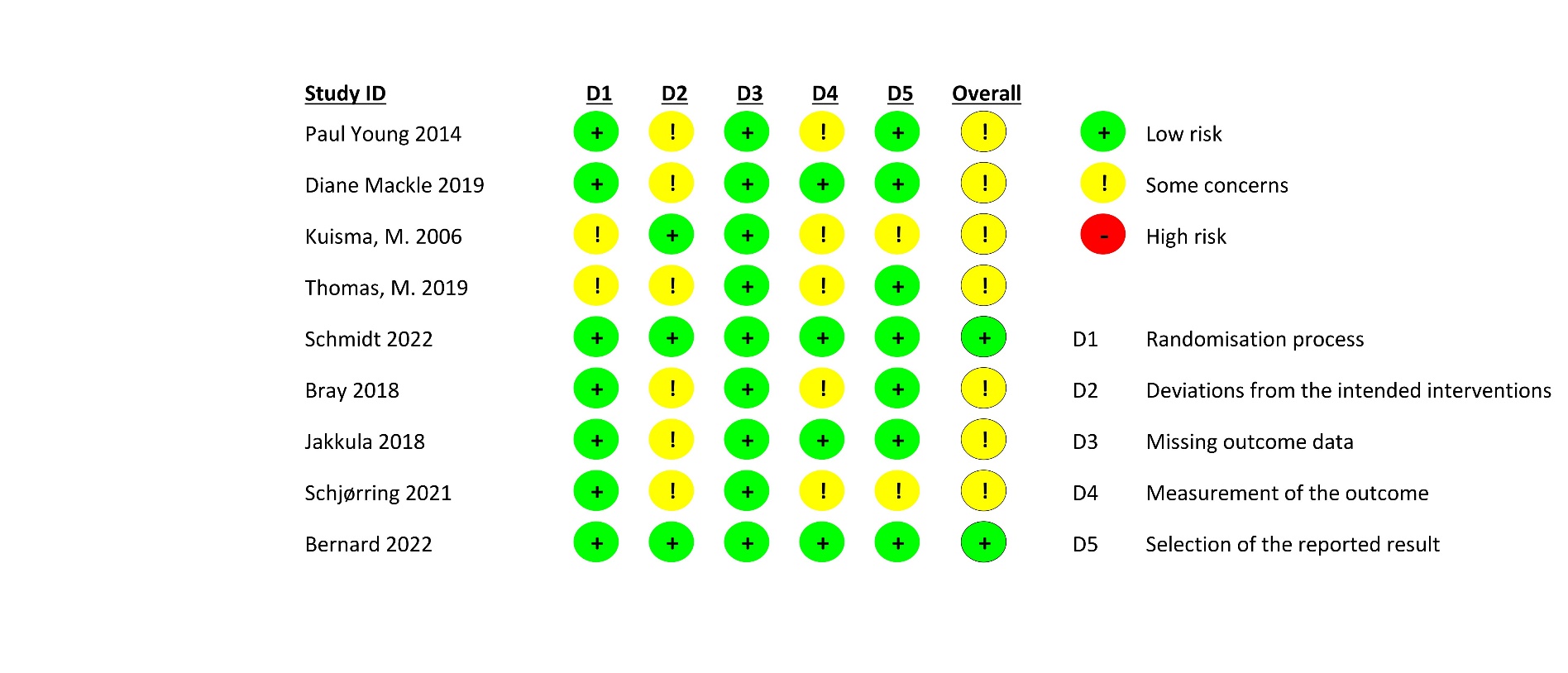

Supplement: Supplementary data 1 [file mmc1.docx]
